# The Story of the Insane from Year to Year

**Published:** 1904-12-10

**Authors:** 


### THE STORY OF THE INSANE FROM YEAR TO YEAR.

Seventeenth Annual Series.

### MONMOUTHSHIRE ASYLUM.

At the beginning of the year 1,111 patients were in the asylum, and at the end of it the numbers had fallen to 900. There were 167 first admissions, 53 readmissions, 86 discharged recovered, 33 discharged relieved, 217 not improved, and 95 died. The total number under treatment was 1,325, and the average daily number resident was 950. The recoveries were 39 per cent. of the admissions, and the deaths were 9·9 per cent. of the average number resident. Of the year's deaths 32 were caused by cerebral and spinal diseases, 10 by consumption, 11 by other lung diseases, and 1 by colitis. Of the year's admissions only 9 were supposed to have had the insanity due to moral causes, and bodily disease comes first among the physical causes with 74, heredity follows with 43, and intemperance in drink for only 13. The latter number is so small for a total of 220 admissions that we are inclined to believe that the statements in the admission papers cannot be quite accurate. Relatives of patients often try to conceal the drink habit. The decrease which took place during the year in the population of the asylum was owing to the removal of 228 patients who had been chargeable to Brecon and Radnor to their own asylum at Talgarth. Dr. Glendinning says that "with the exception of two sporadic cases of typhoid fever and a few cases of erysipelas there was no disease of an epidemic or contagious character," but nevertheless the death-table shows one case of colitis. The committee speak of the "highly satisfactory manner in which Dr. Glendinning" has discharged his duties. The weekly cost of maintenance per head is 8s. 2d. Out-counties pay 14s. and 16s. Private patients apparently from 21s. to 30s. We do not think the former sum (21s.) should ever be exceeded in county asylums.

